# GWAS in the SIGNAL/PHARE clinical cohort restricts the association between the *FGFR2* locus and estrogen receptor status to HER2-negative breast cancer patients

**DOI:** 10.18632/oncotarget.12669

**Published:** 2016-10-14

**Authors:** David G. Cox, Elsa Curtit, Gilles Romieu, Pierre Fumoleau, Maria Rios, Hervé Bonnefoi, Thomas Bachelot, Patrick Soulié, Christelle Jouannaud, Hugues Bourgeois, Thierry Petit, Isabelle Tennevet, David Assouline, Marie-Christine Mathieu, Jean-Philippe Jacquin, Sandrine Lavau-Denes, Ariane Darut-Jouve, Jean-Marc Ferrero, Carole Tarpin, Christelle Lévy, Valérie Delecroix, Véronique Trillet-Lenoir, Oana Cojocarasu, Jérôme Meunier, Jean-Yves Pierga, Céline Faure-Mercier, Hélène Blanché, Mourad Sahbatou, Anne Boland, Delphine Bacq, Céline Besse, Jean-François Deleuze, Iris Pauporté, Gilles Thomas, Xavier Pivot

**Affiliations:** ^1^ Centre de Recherche en Cancérologie de Lyon, INSERM U1052 - Centre Léon Bérard, 69373 Lyon, France; ^2^ Hôpital Jean-Minjoz, Centre Hospitalier Universitaire, Boulevard Fleming, 25030 Besançon, France; ^3^ Oncologie Sénologie, ICM Institut Régional du Cancer, 34298 Montpellier Cedex, France; ^4^ Georges-François Leclerc, 21000 Dijon, France; ^5^ Institut de Cancérologie de Lorraine - Alexis Vautrin, Département d'Oncologie Médicale, 54511 Vandœuμvre-lès-Nancy Cedex, France; ^6^ Institut Bergonié, Département d'Oncologie Médicale, 33000 Bordeaux, France; ^7^ Centre Léon Bérard, Département de Cancérologie Médicale, Lyon Cedex 08, France; ^8^ Institut de Cancérologie de l'Ouest, Service Oncologie Médicale, 49993 Angers Cedex 09, France; ^9^ Institut Jean Godinot, Service Oncologie Médicale, 51056 Reims Cedex, France; ^10^ Clinique Victor Hugo-Centre Jean Bernard, 72015 Le Mans Cedex 2, France; ^11^ Centre Paul Strauss, Service d'Oncologie Médicale, 67065 Strasbourg Cedex, France; ^12^ Centre Henri Becquerel, rue d'Amiens, 76038 Rouen, France; ^13^ Institut Daniel Hollard, Service Oncologie Médicale, 38028 Grenoble Cedex 01, France; ^14^ Institut Gustave Roussy, Comité de Pathologie Mammaire, 94805 Villejuif Cedex, France; ^15^ Institut de Cancérologie Lucien Neuwirth, Service Oncologie Médicale, 42270 Saint Priest en Jarez, France; ^16^ Centre Hospitalier de Limoges, Service d'Oncologie Médicale, 87042 Limoges Cedex, France; ^17^ Clinique Drévon, Centre d'Oncologie et de Radiothérapie du Parc, 21000 Dijon, France; ^18^ Centre Antoine Lacassagne, Département Oncologie Médicale, 06189 Nice Cedex 02, France; ^19^ Institut Paoli-Calmettes, Département d'Oncologie Médicale, 13009 Marseille, France; ^20^ Centre François Baclesse, 14076 Caen Cedex 5, France; ^21^ Pôle Mutualiste, Service Oncologie Médicale, 44606 Saint Nazaire, France; ^22^ Centre Hospitalier Lyon Sud, Service d'Oncologie Médicale, 69495 Pierre-Benite cedex, France; ^23^ Centre Hospitalier Le Mans, Service d'Onco-Hématologie et Médecine interne, 72037 Le Mans Cedex, France; ^24^ Centre Hospitalier Régional d'Orléans, Service d'Oncologie Médicale, 45032 Orleans Cedex 1, France; ^25^ Institut Curie, Department of Medical Oncology, 75248 Paris Cedex 05, France; ^26^ Institut National du Cancer, Direction de la Recherche, 92513 Boulogne-Billancourt, France; ^27^ Fondation Jean Dausset, Centre d'Etudes du Polymorphisme Humain, 75010 Paris, France; ^28^ Centre National du Génotypage, Institut de Génomique, CEA, CP 5721, 91057 Evry Cedex, France; ^29^ Synergie Lyon Cancer, Centre Léon Bérard, Lyon Cedex 08, France

**Keywords:** breast, estrogen receptor, HER2, association, GWAS

## Abstract

Genetic polymorphisms are associated with breast cancer risk. Clinical and epidemiological observations suggest that clinical characteristics of breast cancer, such as estrogen receptor or HER2 status, are also influenced by hereditary factors. To identify genetic variants associated with pathological characteristics of breast cancer patients, a Genome Wide Association Study was performed in a cohort of 9365 women from the French nationwide SIGNAL/PHARE studies (NCT00381901/RECF1098). Strong association between the *FGFR2* locus and ER status of breast cancer patients was observed (ER-positive n=6211, ER-negative n=2516; rs3135718 OR=1.34 p=5.46×10^−12^). This association was limited to patients with HER2-negative tumors (ER-positive n=4267, ER-negative n=1185; rs3135724 OR=1.85 p=1.16×10^−11^). The *FGFR2* locus is known to be associated with breast cancer risk. This study provides sound evidence for an association between variants in the *FGFR2* locus and ER status among breast cancer patients, particularly among patients with HER2-negative disease. This refinement of the association between *FGFR2* variants and ER-status to HER2-negative disease provides novel insight to potential biological and clinical influence of genetic polymorphisms on breast tumors.

## INTRODUCTION

Since the completion of the Human Genome Project, the Genome Wide Association Scan (GWAS) has become the tool of choice for the detection of associations between disease risk and common genetic variation. The first breast cancer risk variants identified in the GWAS era were in the *FGFR2* locus [1,2].

Further analyses, mainly in case-control and prospective cohorts, have reinforced this association as well as identified over 90 additional breast cancer risk loci [3]. GWAS studies with cases selected based on the estrogen receptor (ER) status of their tumors, and control subjects not affected by breast cancer, have shown divergent associations between ER+ and ER- tumors. In these analyses, variants in *FGFR2* are more strongly associated with ER+ disease [4–14], as opposed to ER- disease, when comparing cases to healthy controls. Few single studies, however, have sufficient detail or sample size to carry out case-only analyses to further explore the relationship between genetic variants and disease characteristics, particularly with respect to amplification of the *HER2* gene. Therefore analyses by subtype are often secondary, based on findings of the primary analyses of overall breast cancer risk. Furthermore, these studies are now carried out in large consortia with the potential for heterogeneity in definitions of various case characteristics, particularly ER and HER2 status.

For example, Broeks et al. [13] examined the association between low penetrance breast cancer loci and specific breast tumor subtypes in the context of the Breast Cancer Association Consortium (BCAC). rs2981582 in the *FGFR2* locus was significantly associated with ER+/PR+/HER2- breast cancer (n_cases_=7201, p = 2.2 × 10^−29^), less so with ER+/PR+/HER2+ cases (n_cases_=996, p=5.5×10^−4^), and no association was observed with triple negative breast cancer (n_cases_=1480, p=0.841) or ER-/PR-/HER2+ breast cancer (n_cases_=627, p=0.396). A case-only comparison of HER2 status was carried out within ER+/PR+ and ER-/PR- groups, and neither showed any association (p=0.23 and 0.15, respectively).

In the present study, a case-only GWAS approach was used to study differences in the distribution of variants between breast cancer cases in a large, multi-center study with centralized data collection and handling, the SIGNAL/PHARE case-cohorts (NCT00381901/RECF1098).

## RESULTS

Genotype data was generated from 9365 SIGNAL/PHARE participants. All subjects had greater than 95% genotyping success rate. 26 pairs of individuals were identified with Identity by State (IBS) > 30%, with the subject having the most complete genotype data from each pair retained for analyses. 551 further individuals were excluded from the present study due to PCA analyses. Finally, 61 subjects with missing clinical data were excluded. A total of 8727 patients including 2516 patients with ER- breast cancer were analyzed. Furthermore, 5452 patients had HER2-negative breast cancer, of which 1185 were ER-.

**Figure 1:**
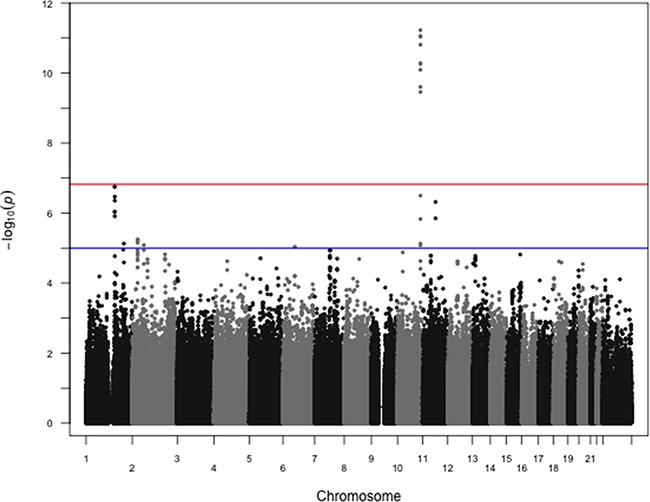
Manhattan plot of associations between SNPs and ER status overall P-values from logistic regression comparing estrogen receptor positive cases to estrogen receptor negative cases, controlling for age at diagnosis and first two principal components, are shown. rs3135718 on chromosome 10 at the *FGFR2* locus shows the strongest association. 914144 SNPs were included in these analyses, with 6211 ER+ and 2516 ER- cases. The red horizontal line corresponds to the empirical significance threshold of 1.48×10^−7^, while the blue horizontal line corresponds to an arbitrary level of 1.0×10^−5^. The inflation factor (λ) for these analyses is 1.02.

**Figure 2:**
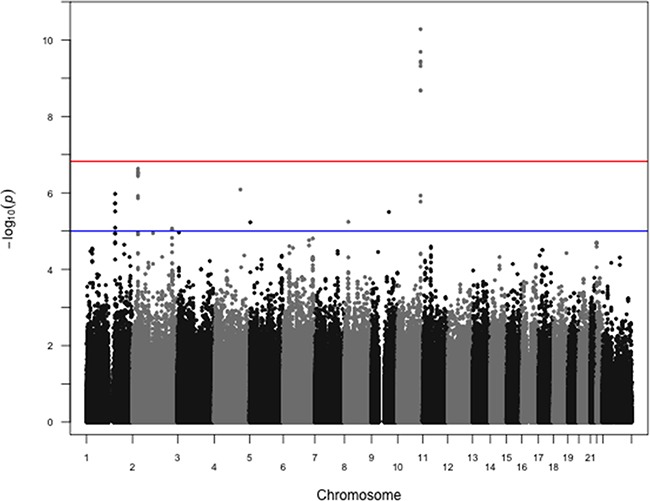
Manhattan plot of associations between SNPs and ER status restricted to HER2− cases P-values from logistic regression comparing estrogen receptor positive cases to estrogen receptor negative cases restricted to HER2− cases, controlling for age at diagnosis and first two principal components, are shown. rs2981578 on chromosome 10 at the *FGFR2* locus shows the strongest association. The same 914144 SNPs were included in these analyses, with 4267 HER2-/ER+ and 1185 HER2-/ER- cases. The red horizontal line corresponds to the empirical significance threshold of 1.48×10^−7^, while the blue horizontal line corresponds to an arbitrary level of 1.0×10^−5^. The inflation factor (λ) for these analyses is 1.02.

The search for variants associated with ER status showed only one region with a highly significant association, corresponding to *FGFR2* (best p-value for rs3135718 p-value=6.0×10^−12^, Figure 1 and Supplementary Figure S1). Restricting our analyses to HER2-negative cases found that associations between variants at the *FGFR2* locus remained significant at the genome-wide level (best p-value for rs3135724 = 5.2×10^−11^, Figure 2). Among HER2-positive tumors, the lowest p-value in the *FGFR2* locus for the association with ER status was found for rs2981578 (p = 3.3×10^−4^ Table 1). The four variants in Table 1 were chosen to highlight the difference in associations between HER2+ and HER2− patients. Despite the smaller sample size among HER2-positive cases, this study has nearly 100% power to detect a per-allele OR = 1.8 as observed among the HER2-negative tumors, and greater than 80% power to detect a per-allele OR ≈ 1.3. The observed direction of the association was consistent with observations in prior case-control studies, with for example the C allele of rs3135718 being more frequently reported among women with ER+ tumors.

**Table 1:** Selected variants at the *FGFR2* locus and ER status among breast cancer cases

		Overall		HER2+		HER2−	
SNP	I/G* (Rsq, Quality)	OR (95% CI)	p	OR (95% CI)	p	OR (95% CI)	p
rs3135718	I (0.64, 0.89)	1.33 (1.23 - 1.45	6.0×10-12	1.19 (1.04 - 1.35)	7.9×10-3	1.47 (1.30 - 1.64)	2.0×10-10
rs3135724	I (0.41, 0.84)	1.51 (1.33 - 1.69)	8.1×10-11	1.18 (0.97 - 1.41)	9.3×10-2	1.79 (1.49 - 2.13)	5.2×10-11
rs2981578	G (NA, NA)	1.24 (1.16-1.32)	3.5×10-10	1.20 (1.09-1.33)	3.3×10-4	1.26 (1.14-1.38)	1.7×10-6
rs2981579	G (NA, NA)	1.25 (1.16 - 1.33)	5.5×10-11	1.15 (1.03 - 1.27)	9.2×10-3	1.33 (1.20 - 1.47)	2.1×10-9

* Imputed (I) or genotyped (G). Values reported from MACH output

As mentioned previously, variants in the *FGFR2* locus were the first identified via GWAS with respect to breast cancer risk. The most recent fine-mapping effort of the *FGFR2* locus explored functional variants, and identified three separate independent sets of correlated highly associated variants (ICHAVs [18]). In the present analyses restricted to HER2-negative tumors, rs3135724 was the SNP with the strongest association for ER status. These data included rs2981579 and rs2981578, from ICHAVs 1 and 3 respectively (Table 1). Unfortunately, rs45631563 from ICHAV 2 was not included, and no SNPs showed significant linkage disequilibrium with this marker in the current 1000 genomes data (http://1000genomes.org accessed July 8, 2015). Therefore additional analyses were carried out including rs3135724, rs2981579, and rs2981578 in the same logistic regression model. In our analyses of HER2− breast cancer, we found no evidence for independent association between these variants and tumor ER status (data not shown).

## DISCUSSION

The identification of variants associated with specific molecular subtypes of breast cancer was a primary aim of the prospective SIGNAL/PHARE cohort. In this high-powered GWAS performed in a case-cohort of breast cancer patients with detailed clinical data, further information with respect to variants in the *FGFR2* locus and their influence on breast cancer were provided, particularly regarding tumor ER status. In addition, the association between variants in *FGFR2* and ER status in breast cancer was stronger among patients with HER2− tumors. While not including an independent validation set is a drawback of our analyses, the large sample size allowed us to have sufficient power to fully define this association, and the p-values obtained were well below empirical estimations of significance thresholds (1.48×10^−7^) as well as the generic GWAS significance threshold of 5×10^−8^.

Our hypothesis is that genetic variants that are associated with molecular subtypes will provide novel insights regarding disease etiology, and may lead to further developments regarding disease prevention and treatment. As our main focus was the construction of a clinical cohort, we have focused on collecting information with respect to histo-pathology and treatments, and patient follow-up. Therefore, we have not collected detailed information regarding epidemiological data such as body-mass index, reproductive history and menopausal status, or family history/BRCA mutations. The participants have been given a self-administered questionnaire with some of these variables, but as this questionnaire was administered after cancer diagnosis, we have chosen to not exploit these data at this time.

We have focused on the *FGFR2* locus, which showed the strongest association with ER status, particularly among HER- breast cancer patients. There is growing evidence that genetic variants may be more strongly associated with specific breast cancer subtypes. For the most part, these analyses are extensions of current prospective cohort and case-control analyses. For example, recent analyses by Michailidou et al. [3] included stratification by estrogen receptor status for the 77 variants included in their polygenic risk score. A number of these variants showed differential associations with respect to estrogen receptor status. However as the authors state in their discussion, the number of estrogen receptor negative cases made accurately determining risk estimates difficult for this cancer subtype. Future analyses in our case-cohort will investigate other variants previously shown to influence breast cancer subtype.

A potential limitation of our study is the use of an internal imputation process, as opposed to imputing to the commonly used 1000 Genomes data or the Michigan Imputation Server. As highlighted in the Methods this was our original study design prior to the availability of these resources. We have continued with this approach in order to avoid any potential population differences with respect to linkage disequilibrium between our population of French breast cancer cases and the populations that provided data for publicly available resources. This approach leads to a lower number of variants on the absolute scale, meaning that we may be unable to detect any additional variants not captured through genotyping with the Illumina Omni5, which captures over 80% of common variants among Caucasian populations, and strict quality filtering of data (See Methods section).

For aspects of response to treatment, SIGNAL/PHARE has not yet accrued enough follow-up to fully explore the implication of variants on patient's outcome. This will be of course an obvious next step of our analyses, particularly as pertains to response to hormone therapy and *FGFR2* variants in ER+/HER- breast cancer patients.

In conclusion, we further refine the influence of variants in the *FGFR2* locus with respect to molecular characteristics of breast tumors, in that they are more strongly associated with estrogen receptor status among cancers without amplification of the *HER2* gene.

## METHODS

PHARE was a randomized phase 3 clinical trial comparing 6- and 12-month trastuzumab adjuvant exposure [15], which included a subset of 1,430 HER2-positive breast cancer cases with DNA available for GWAS analyses. SIGNAL was a prospective cohort specifically designed for GWAS analyses of 8,406 early breast cancer patients, enrolled at the time of the adjuvant chemotherapy from June 2009 to December 2013. The combined data set, the PHARE/SIGNAL study, included 9,365 breast cancer patients. Clinical and pathological characteristics were prospectively collected using standardized forms, and centralized at the French National Cancer Institute (INCa). For both studies, patients provided blood samples that were centralized at the Centre d'Etude du Polymorphisme Humain (CEPH) in Paris, France, for DNA extraction using standard protocols. Genotyping was carried out at the Centre National de Génotypage (CNG) in Evry, France.

The original study plan called for a two-staged genotyping strategy using only study participants. This approach aimed at reducing the potential that population structure in French breast cancer cases would influence imputation, while maximizing the proportion of the genome covered. Briefly, all cases were genotyped using the Illumina HumanCore Exome chip set, composed of over 264000 variants for a “GWAS Backbone” and over 244000 “exome-centered” variants. Variants were filtered based on completion rates (<95% SNP success, N = 8122), departure from Hardy-Weinberg Equilibrium (HWE p<0.001, N = 20357), and low minor allele frequency (MAF<0.001, N=200628). Principal Components Analysis (PCA) and k-means were then used to characterize the ancestry of the participants and only the main cluster of European individuals was included in the present analysis, to reduce risk of population stratification (See Supplementary Figure S2). A random subset of 1449 individuals from the main “European” cluster was selected for genotyping using the Illumina Omni5 chip set, composed of over 4M variants (See Supplementary Figure S2). Complete (SNP success = 100%, N=2049173) Omni5 data were then filtered using similar cutoffs as the HumanCore Exome data, specifically HWE (p<0.001, N=91018) were then used to impute missing genotypes from the remaining subjects genotyped using the HumanCore Exome array. SNPs with imputation quality score < 30% were excluded from analyses (N=783416), and finally variants with a MAF < 0.01 were excluded (N=82847). A total of 914144 SNPs were included in the GWAS analyses. Standard GWAS logistic regressions were carried out using the ProbABEL package [16]. Age at diagnosis and the first two principal components were included in regression analyses.

Genome-wide significance levels were estimated using the effective number of tests based on linkage disequilibrium between all markers used in our population through the SimpleM function in R [17]. The number of effective markers is estimated at 345906, corresponding to a Bonferroni-corrected p-value threshold of 1.48×10^−7^.

## SUPPLEMENTARY MATERIALS FIGURES


