# Supplementary material for: GWAS in the SIGNAL/PHARE clinical cohort restricts the association between the FGFR2 locus and estrogen receptor status to HER2-negative breast cancer patients
Source: Oncotarget. 2016 Oct 14;7(47):77358–64. doi: 10.18632/oncotarget.12669 (PMC5363591; doi:10.18632/oncotarget.12669)
Supplement: Supplementary file 1 [file oncotarget-07-77358-s001.pdf]

GWAS in the SIGNAL/PHARE clinical cohort restricts the association between the *FGFR2* locus and estrogen receptor status to HER2-negative breast cancer patients

SUPPLEMENTARY FIGURES

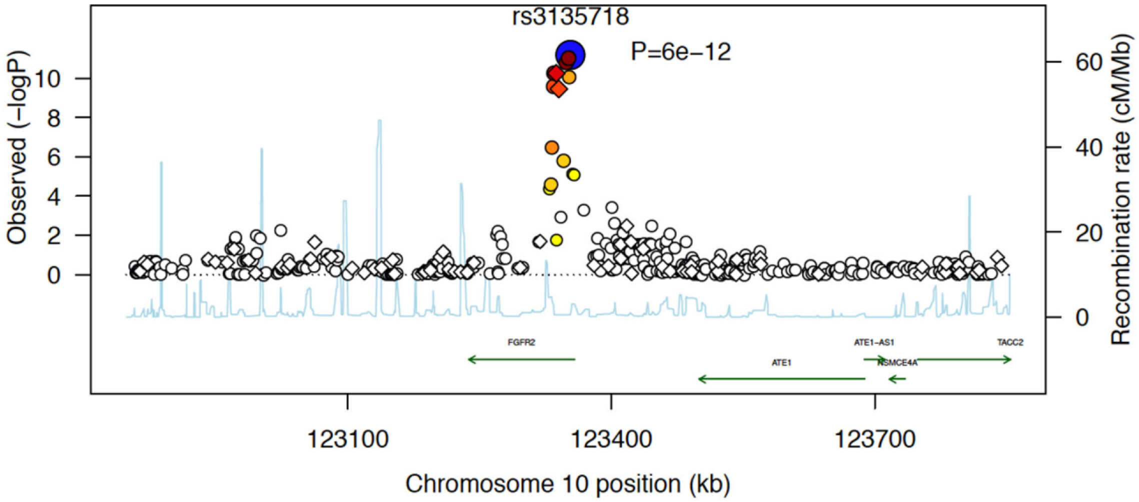

Supplementary Figure S1: Locuszoom plot of chromosome 10 around the FGFR2 locus. Circles represent imputed SNPs, diamonds represent genotyped SNPs. Observed p-value is plotted along the left Y axis, recombination rate along the right Y axis. Shading from purple to yellow in filled shapes represents linkage disequilibrium with the highlighted SNP, in this case is rs3135718.

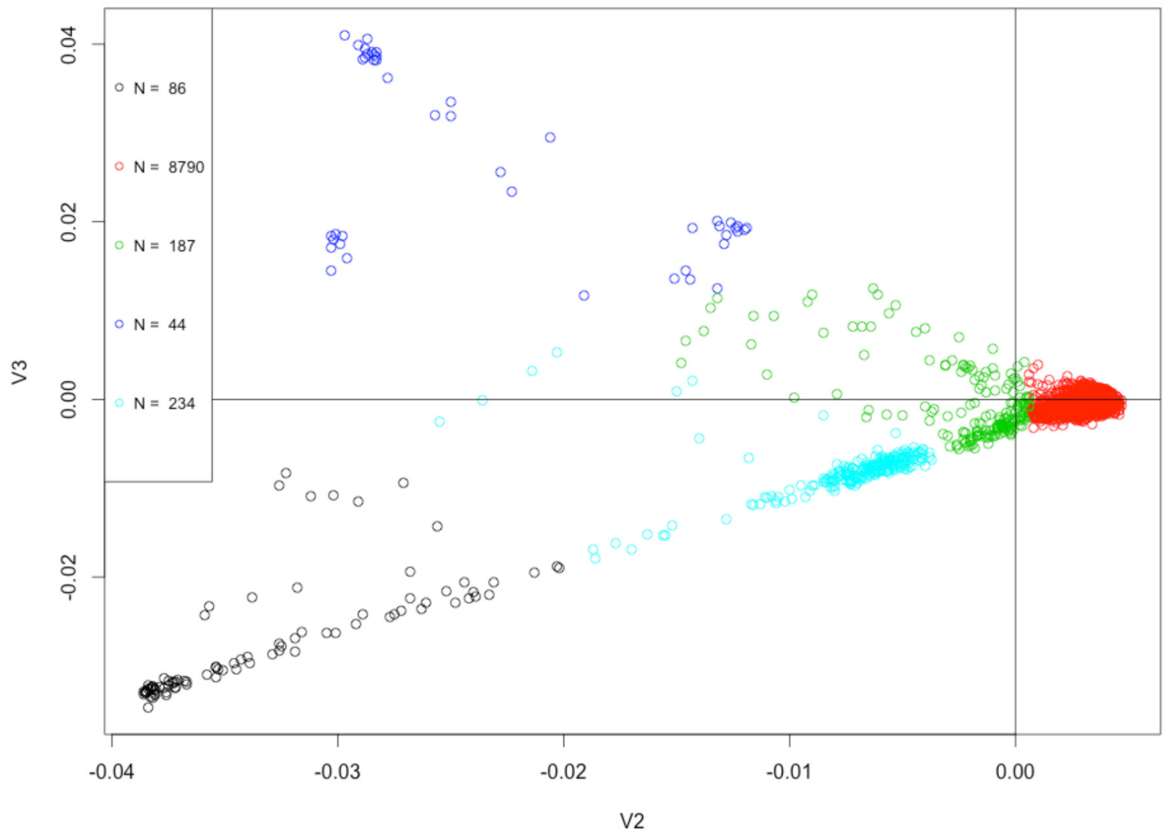

Supplementary Figure S2: Principal components and K-means analyses of genotyping data from the HumanCore Exome data in the combined SIGNAL/PHARE cohorts.
